# Timing of recurrence after treatment of pulmonary TB

**DOI:** 10.5588/ijtldopen.24.0222

**Published:** 2024-10-01

**Authors:** C.T. Mithunage, D.W. Denning

**Affiliations:** Manchester Fungal Infection Group, Faculty of Biology, Medicine and Health, Manchester Academic Health Science Centre, University of Manchester, Manchester, UK.

**Keywords:** bronchiectasis, chronic pulmonary aspergillosis, HIV, reinfection, recurrence

## Abstract

Pulmonary TB (PTB) may recur due to reinfection or relapse after initial successful treatment. Based on microbiologically documented cases, we searched Embase, PubMed, Web of Science, and Medline for PTB recurrence. The timeframe of overall recurrences, relapse, reinfection, and risk factors were assessed. We compared the time to recurrence, relapse, and reinfection from treatment completion and plotted this using Kaplan–Meier curves. This systematic review included 23 articles describing 2,153 PTB recurrences in 75,224 treated people across all continents. Genotyping data to distinguish relapse from reinfection was available for 402 recurrences. The cumulative recurrence percentage was 2.9% over 5 years, and the median time for recurrence was 18 months (95% CI 16.99–19.0). Most recurrences (93%) were in HIV-negative people. Relapse occurred earlier than reinfection at 12 months (95% CI 10.86–13.14) vs 24 months (95% CI 21.61–26.39) (*P* < 0.001, χ^2^ 59.89). In low TB burden settings, recurrences were mainly caused by relapse (85%), whereas in high-burden settings, relapses comprised 56% of recurrences. Recurrences occurred slightly earlier in HIV-positive patients (*P* = 0.038, χ^2^ 4.30). The emergence of resistance to one or more first-line anti-TB agents was documented in 40 of 421 cases (9.5%). Early recurrences are mainly relapses, while late recurrences are mainly reinfections.

## RÉSUMÉ

La TB pulmonaire (TBP) peut récidiver en raison d'une réinfection ou d'une rechute après un premier traitement réussi. En nous basant sur des cas documentés sur le plan microbiologique, nous avons mené une recherche dans les bases de données Embase, PubMed, Web of Science et Medline concernant la récurrence de la TBP. Nous avons évalué le calendrier des récidives globales, des rechutes, des réinfections ainsi que des facteurs de risque associés. De plus, nous avons comparé les délais de récurrence, de rechute et de réinfection à partir de la fin du traitement, en les illustrant à l'aide de courbes de Kaplan-Meier. Cette analyse systématique a examiné 23 études rapportant 2 153 cas de récidive de TBP parmi 75 224 individus traités à l'échelle mondiale. Des informations de génotypage permettant de différencier les rechutes des réinfections étaient accessibles pour 402 cas de récidive. Le taux cumulé de récidive s'élevait à 2,9% sur une période de 5 ans, avec un délai médian de récidive de 18 mois (IC à 95% 16,99–19,0). La majorité des récidives (93%) concernait des individus séronégatifs. Les rechutes se sont produites plus rapidement que les réinfections, à 12 mois (IC 95% 10,86–13,14) contre 24 mois (IC 95% 21,61–26,39 ; *P* < 0,001 ; χ^2^ 59,89). Dans les environnements où le fardeau de la TB est faible, les récidives étaient principalement attribuées à des rechutes (85%), tandis que dans les contextes à fardeau élevé, les rechutes représentaient 56% des récidives. Les récidives se sont manifestées légèrement plus tôt chez les patients séropositifs (*P* = 0,038, χ^2^ 4,30). L'apparition d'une résistance à un ou plusieurs médicaments anti-TB de première ligne a été observée dans 40 cas sur 421, soit 9,5%. Les récidives précoces sont majoritairement des rechutes, tandis que les récidives tardives sont principalement dues à des réinfections.

TB is the second leading infectious cause of death after COVID-19 worldwide. In 2021, an estimated 10.6 million people were diagnosed with TB globally, and 83% (8.8 million) had pulmonary TB (PTB).^1^ Recurrent TB is an additional episode of TB disease in a patient previously adequately treated and cured. Recurrent TB is categorised into two entities depending on the strain of the *Mycobacterium tuberculosis* causing the subsequent episode. Relapse is the endogenous re-activation of the previous infection with the same *M. tuberculosis* strain, which was not eliminated during the treatment.^2^ Certain bacterial factors, such as phenotypic drug tolerance in bacterial persisters and other host factors, such as pharmacogenetic characteristics, facilitate endogenous re-activation.^3,4^ Exogenous reinfection is a new infection caused by a different strain than the first episode.

Most studies on recurrent TB have reported a specific proportion who have recurrence over a follow-up time of 2 years or sometimes 5 years, without detailing the specific timeframe of recurrence of PTB. In this study, our primary purpose was to determine the timeframe of PTB recurrence, particularly according to the type of recurrence, to alert clinicians about the misidentification of conditions with a similar presentation.

## METHODS

The Preferred Reporting Items for Systematic Reviews and Meta-Analyses (PRISMA) guidelines were followed throughout the process of conducting and presenting this systematic review. This study did not require ethical approval or informed consent because it was a systematic review of previously published studies. The study was registered in PROSPERO in July 2022 (registration number: CRD42023433818).

### Search strategy and study selection

A comprehensive literature search was performed through PubMed, Web of Science, Embase, and Medline databases as the source of literature for this study using the following keywords, title/abstracts, and Medical Subject Headings (MeSH) terms: (pulmonary tuberculosis or pulmonary TB or PTB) AND (recurrence or re-activation or relapse or reinfection) AND (frequency or timeframe) for articles published from 1967 to August 2023. EndNote tm20 was utilised to manage the bibliography received from the search during the entire review procedure. One author independently screened the search results based on the title and abstract, and another helped confirm eligibility based on the inclusion criteria. Many articles retrieved were in foreign languages such as Russian, Polish, Romanian, and Chinese.

### Inclusion and exclusion criteria

For inclusion, studies had to meet all the following criteria: 1) confirmed initial PTB with smear, culture, polymerase chain reaction (PCR), or GeneXpert (nucleic acid amplification test for simultaneous rapid TB diagnosis and rapid antibiotic sensitivity test); 2) the first episode was treated with WHO-recommended treatment: a 6-month combination regimen consisting of 2 phases; a 2-month phase with isoniazid (INH), rifampicin (RIF), pyrazinamide (PZA), and ethambutol, followed by a 4-month phase of INH and RIF only combination; 3) confirmed second PTB infection with positive smear, culture, PCR, or GeneXpert result; and 4) documented timing of recurrence or relapse after initiation or completion of TB treatment.

The exclusion criteria were as follows: 1) extrapulmonary TB; 2) single-case studies or study sample size less than 10; 3) paediatric TB (less than 13 years); 4) no information on the timeframe of recurrence, relapse, or reinfection; 5) recurrent PTB solely based on radiology or clinical findings; and 5) multidrug-resistant PTB.

### Data extraction

The same author extracted all relevant data on the main characteristics: author, published year, study country and period, design, study population, sample size, methods used, inclusion and exclusion criteria, case definitions, treatment regimens, recurrence timeframe, number of relapses or reinfections, and HIV or diabetes status. The data extracted from full texts of included studies was added into a standardised MS Excel (Microsoft, Seattle, WA, USA) sheet.

### Quality assessment

We performed quality assessments using the Newcastle-Ottawa Scale (NOS) to assess the methodological quality of the included articles. The quality of a study was given a score of 0–3 for low quality, 4–6 for moderate quality, and 7–8 for high quality. During the evaluation of the quality of the included studies, any uncertainties were discussed with the other researcher, and a score was agreed upon.

### Data analysis

In our systematic review, recurrent TB was defined as a bacteriologically confirmed PTB episode recorded after the completion of standard treatment preceded by an initially successfully treated PTB episode. Time to recurrence, relapse, and reinfection was calculated from the completion of treatment, not from the initiation of treatment or diagnosis of PTB.

The timing of recurrence, relapse, or reinfection was recorded in most articles in either days or months. Some studies documented it based on yearly or 2-yearly numbers of cases. Except for four articles, others documented the recurrence time since treatment completion. Three articles stated the duration of treatment was 6 months; thus, by deducting 6 months, the time duration for recurrence was calculated. One article did not mention the exact duration of treatment, so we assumed the treatment duration was 6 months. Finally, 15 articles were grouped and presented 6-monthly recurrences, six articles presented yearly recurrences, and two articles presented 2 yearly recurrences. Except for two articles, others did not mention the number of deaths or number lost to follow-up during the monitoring period for recurrences or relapses. We assumed none of the patients died or were lost to follow-up for the other 21 articles. Therefore, when the number of patients at risk was calculated at the end of each time interval, only the cumulative recurrences were deducted (one of the limitations of the study). The cumulative percentage of recurrence was calculated and plotted.

We conducted a subgroup analysis utilising 13 studies which focused on distinguishing between relapse and reinfection of fully susceptible PTB. According to the WHO classification, this subgroup conducted studies in countries with low and high burdens. The high-incidence countries included China, India, Malawi, Uganda, and South Africa (eight studies), and the low-incidence countries included Australia, the United States, Canada, Spain, and Italy (five studies). For this subgroup analysis of relapse or reinfection, 15,793 patients with genotyping data available were considered. A subsidiary subgroup analysis was then conducted to differentiate between relapse and reinfection in HIV-positive and HIV-negative patients, using five studies.

### Statistical analysis

The Kaplan–Meier curves were generated in SPSS v29.0 software (IBM Corp, Armonk, NY, USA) to describe and compare the time to recurrence and type of recurrence (relapse or reinfection) in the general population. χ^2^ tests were used to compare the difference between relapse and reinfection. Similarly, log-rank tests and χ^2^ tests were used to compare the time to recurrence between HIV status and type of recurrence. All statistical analyses were performed using SPSS software. Significance (*P*) < 0.05 was considered statistically significant, and 95% confidence intervals (CIs) were generated.

## RESULTS

### Search results

A total of 2,627 articles were initially screened according to the search terms. Then, we excluded 275 citations for duplication. Another 18 citations published in other languages (Romanian, Polish, Chinese) were also excluded. After screening titles and abstracts, 2,082 articles were removed. Twelve articles in Russian were checked for suitability by a colleague, and none matched our inclusion criteria. Two hundred and thirty-five citations were removed for lack of details on timeframe, unreliable diagnostic methods, paediatric TB, or insufficient data. Therefore, we retrieved 23 studies that met our inclusion criteria (Supplementary Figure S1).

The 23 cohort studies included are described in the Table. The geographical study areas included Europe (*n* = 3), the Americas (*n* = 3), Africa (*n* = 6), Asia (*n* = 10), and one in Australia. Thirteen articles were from high TB burden countries.

**Table.** Characteristics of the studies included in the systematic review (all references listed in Supplementary Data).

First author	Publication year	Duration	Country	Study design	Sample size	Recurrences	Genotyping availability	Data on resistance?*
					*n*			*n/N* (%)
Caminero et al.	2001	5 years	Canary island	Retrospective cohort study	962	18	N/A	3/18 (17)
Cacho et al.	2007	12 years	Spain	Retrospective descriptive study	633	8	N/A	No
Johnson et al.	2000	2 years	Uganda	Prospective epidemiological study	225	14	N/A	No
Uys et al.	2015	12 years	South Africa	Retrospective cohort study	943	163	57	No
Pascopella et al.	2011	14 years	USA	Retrospective cohort study	26,004	148	N/A	14/147 (10)
Dobler et al.	2009	12 years	Australia	Retrospective cohort study	3,731	15	15	1/15 (7)
Sonnenberg et al.	2001	30 months	South Africa	Retrospective cohort study	326	65	39	4/65 (6)
Bandera et al.	2001	4 years	Italy	Epidemiology study	2,127	32	N/A	2/32 (6)
Crampin et al.	2010	8 years	Malawi	Retrospective cohort study	584	53	39	1/26 (4)
Jasmer et al.	2004	7 years	USA & Canada	Retrospective clinical trial	1,244	79	75	1/14 (7)
Marx et al.	2014	12 years	South Africa	Retrospective cohort study	1,869	203	130	No
Glynn et al.	2004	5 years	Malawi	Retrospective cohort study	1,039	21	21	2/18 (11)
Chang et al.	2004	3 years	China	Nested case-control study	12,183	113	N/A	No
Hung et al.	2015	12 years	Taiwan	Nested case-control study	4041	152	N/A	No
Narayanan et al.	2010	6 years	South India	Retrospective cohort study	387	44	25	No
Thomas et al.	2005	18 months	South India	Prospective cohort study	503	62	N/A	10/49 (20)
Vieira et al.	2016	10 years	Brazil	Retrospective epidemiological study	963	47	N/A	No
Moosazadeh et al.	2015	9 years	Iran	Retrospective cohort study	1,271	106	N/A	No
Youn et al.	2022	11 years	South Korea	Retrospective cohort study	2,226	150	N/A	No
Ruan et al.	2021	7 years	China	Longitudinal retrospective study	9,828	479	N/A	No
Shao et al.	2021	6 years	China	Population-based cohort study	1,451	23	23	1/12 (8)
He et al.	2023	7 years	China	Retrospective cohort study	2,141	117	36	1/25 (4)^†^
Pamra et al.	1976	3 years	India	Retrospective cohort study	543	41	N/A	No^‡^

* Data included if susceptibility was tested twice at initial diagnosis and if it relapsed. Reinfection cases not included. Cases included if the relapse episode isolates manifest resistance to any first-line anti-tuberculous agent.

† Two additional patients developed resistance to fluoroquinolones, documented on relapse.

‡ Pre-rifampicin use, therefore excluded.

N/A = not available.

## RESULTS

**Figure 1.**
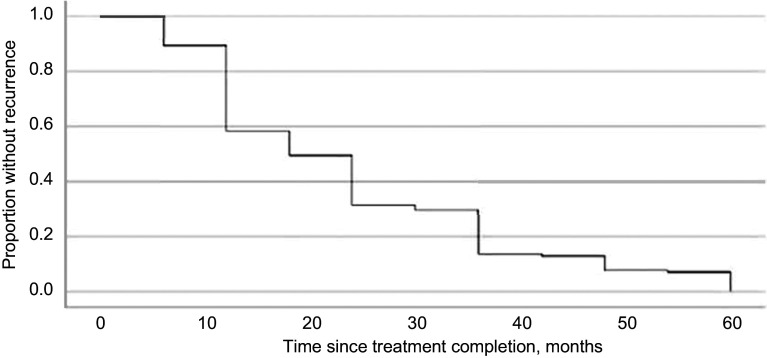
Kaplan-Meier plot of pulmonary TB recurrence.

**Figure 2.**
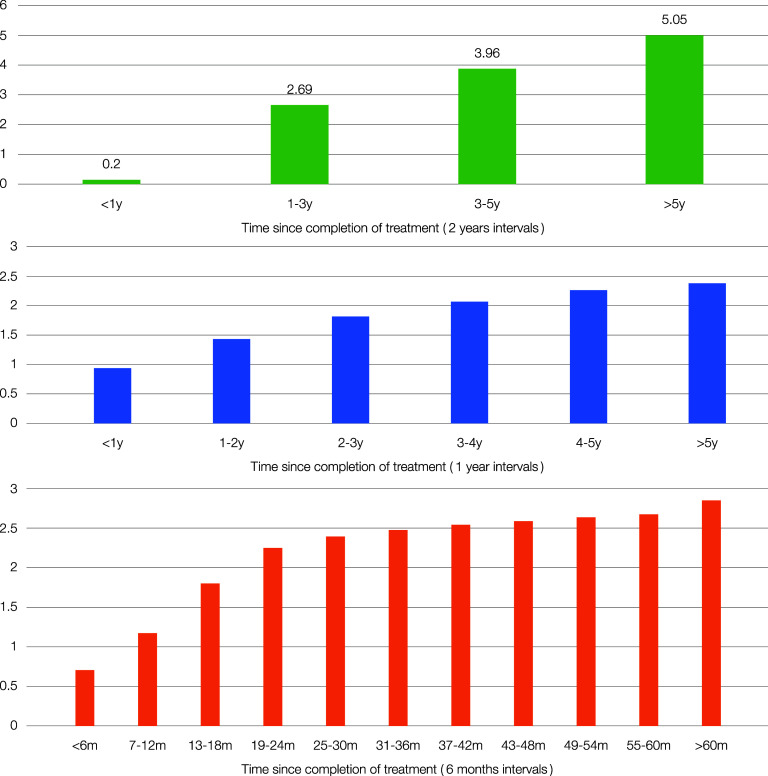
Histogram showing cumulative percentage of recurrences since completion of treatment over 6-monthly, yearly and 2-yearly intervals.

The total number of patients included was 75,224 in these 23 studies, and recurrent TB was documented in 2,153 (2.9%). The median time for recurrence of PTB was 18 months after completion of treatment (95% CI 16.99–19.0) (Figure 1). Cumulative recurrence percentage was plotted according to the time intervals given in the studies. Of the reviewed articles, 15 cases of recurrence were collected in 6-month intervals, where the cumulative recurrence percentage after 5 years of completion of treatment was recorded as 2.9% (Figure 2). An approximately similar value (2.8%) was calculated for the six studies, which documented yearly recurrences (Figure 2). In contrast, significantly higher cumulative percentages (5.1%) were shown by the two articles documenting recurrences 2-yearly (Figure 2). Notably, the cumulative recurrence percentage at the end of 24 months was 2.3%, more than 75% of the overall recurrences.

Among 15,793 patients (*n* = 14 studies) whose genotyping data was available to distinguish between relapse and exogenous reinfection, 652 patients reported recurrent PTB following treatment completion. For 250 recurrences, molecular genotyping results were not available. Of the remaining 402, 270 (67%) were attributed to relapse, while 132 (33%) had acquired exogenous reinfection.

The emergence of resistance to one or more first-line anti-mycobacterial agents was seen overall in 40/421 (9.5%) relapse cases, with individual study data from 4% to 20% (Table). Most emergent resistance was to a single drug, especially INH.

When low-incidence countries were grouped, recurrent TB occurred in 152 (1.7%) of 8,697 PTB patients. Genotyping data for four patients were not available. From 148 patients with genotyping data, 127 (85%) relapses were due to the same *M. tuberculosis* strain as the initial infection, and in 21 (15%), a new *M. tuberculosis* strain was found (reinfection). In contrast, in high-incidence countries, recurrent TB accounted for 500 (7%) of 7,096 PTB cases. Genotyping data to distinguish between relapse and reinfection was not available for 244 recurrences but was for 254 patients, and of these, 143 (56%) had a relapse, and 111 (44%) had exogenous reinfection with a different strain.

**Figure 3.**
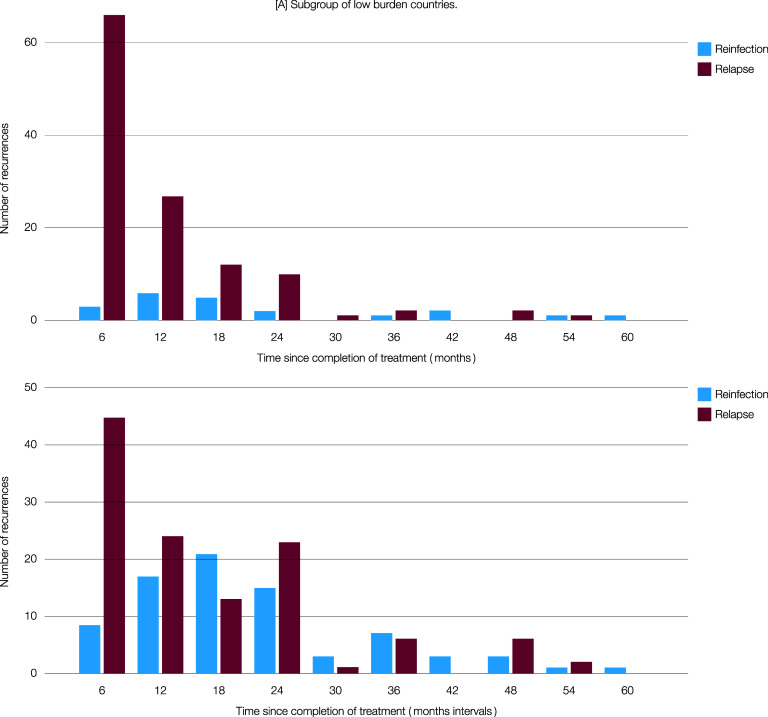
Number of recurrences caused by relapse and reinfection in A. low and B. high burden countries.

The timing of recurrence varied by reinfection or relapse. In the low-burden country subset, recurrent PTB within 24 months after completion of treatment was recorded in 131/148 (88.5%), including 115/127 (90%) with relapse and 16/21 (76%) exogenous reinfections (Figure 3A). Meanwhile, in the high-burden country subset, 165/254 (65%) of recurrences were documented within 24 months of treatment completion, with 105/143 (73%) being relapse and 60/111 (54%) reinfection (Figure 3B). This difference can be explained by relapses being early recurrences, which are predominant in low-burden countries, and by reinfections occurring later, which predominate in high-burden countries.

**Figure 4.**
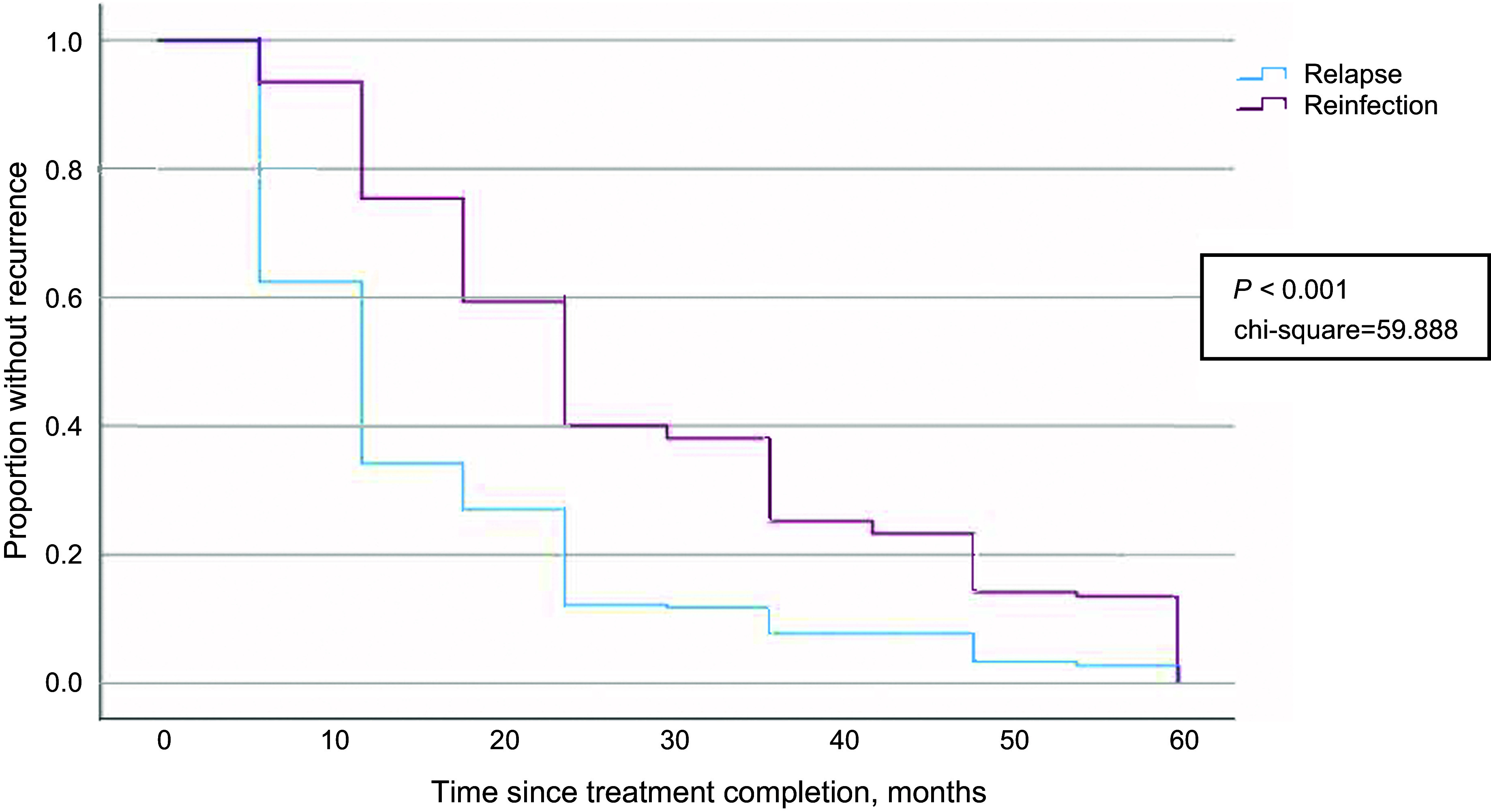
Kaplan-Meier survival estimate for pulmonary TB recurrence. Comparison of time interval between two types of recurrences: relapse and reinfection.

The timing of relapse and reinfection is shown in Figure 4. The median time interval from treatment completion to relapse was 12 months (95% CI 10.858–13.142) in contrast to reinfection, which was twice the time to relapse at 24 months (95% CI 21.609–26.391) (*P* < 0.001, χ^2^ 59.888).

### Impact of HIV infection

Some studies addressed HIV-infected patients using DNA fingerprints from the initial and subsequent episodes of TB; these were available for 192 of 229 recurrences (83.8%). Among 85 HIV-positive patients, 41 (48%) relapses were identified compared to 120 (93%) relapses in the non-HIV patient group (total *n* = 129). In these studies, 52% (44/85) of recurrences in HIV-positive patients were attributable to reinfection vs 7% (9/129) in those who were HIV-negative.

Comparison of the time interval from the end of treatment to recurrence according to HIV status (Supplementary Figure S2A) showed that HIV patients were likely to have a recurrence slightly earlier than HIV-negative patients (*P* = 0.038, χ^2^ 4.303). However, the median time for a recurrence from treatment completion for both HIV-positive and HIV-negative cohorts was the same at 12 months (95% CI 10.57–13.47). Among all recurrences, the time interval from treatment completion to relapse was not different between HIV-positive and HIV-negative people (*P* = 0.280, χ^2^ 1.168) (Figure S2B). The median time to relapse was 6 months for both HIV-positive and HIV-negative groups. Although the median times for reinfection for HIV-positive and HIV-negative people were slightly different at 18 months and 24 months, respectively, this was not significantly different (*P* = 0.117, χ^2^ 2.463) (Supplementary Figure S2C).

## DISCUSSION

From the 23 articles, 75,224 patients with mostly drug-susceptible PTB were included in our systematic review. All the patients had confirmed PTB and completed their treatment successfully. There was a 2.8% cumulative recurrence rate, and the overall median time for recurrence was 18 months, with 88.5% documented within 24 months in low-burden settings. Using available genotyping data to distinguish between relapse and reinfection, we found a relapse rate of 1.7% and a reinfection rate of 0.8% over 5 years.

Countries with a low incidence of TB demonstrate a low PTB recurrence proportion (1.7%), compared to a 7% recurrence rate in higher-incidence countries. Recurrences attributable to relapse predominated in the low-burden setting, while both relapse and reinfection contributed approximately similar proportions in high-burden settings. As expected, these findings mirror single-country studies where recurrences resulting from reinfection were higher in settings with a high TB prevalence,^5–20^ while recurrences mainly were linked to relapse in low-burden countries.^21,22^

The median time for reinfection was 24 months, compared to 12 months for relapse in the overall population. This result differs from that of He et al. in China,^23^ where no significant time difference was found between relapse and reinfection, unlike most other studies.^17,21,23–30^

In those HIV-infected, the proportion of recurrences caused by reinfection and recurrences caused by relapse were approximately similar. In contrast, in the HIV-negative cohort, recurrences were predominantly by relapse. Many of these studies were conducted in Africa, where a higher burden of TB is linked to a higher HIV burden. In contrast, the studies conducted in Europe found no difference between the rates of exogenous PTB reinfection and HIV status.^8,12,28,29,31–38^ Those living in low TB incidence settings have a lesser chance of acquiring a new isolate of *M. tuberculosis*, as demonstrated by many studies.^32,37,39^ HIV-negative patients showed a slightly shorter time interval to recurrence compared to HIV-positive patients, and this was predominantly relapse. However, although this time interval difference was not significantly different, it might have been if more patients had been studied. One study from the late 1990s in South Africa found no difference in the timing of relapse between HIV-positive and HIV-negative patients.^28^ Another article from China assessed the effect of sex, ethnicity (Han and Tu), presence of a pulmonary cavity, multidrug-resistant TB (MDR-TB), and *M. tuberculosis* genetic background (Lineage 2/Lineage 4) against the time interval to relapse.^23^ The study found TB relapse occurs earlier in patients of Tu ethnicity compared to patients of Han ethnicity (*P* < 0.0001). No other significant differences were found.

Among the risk factors for recurrence, diabetes is an important concern in developing countries, where the prevalence of both TB and diabetes is high, and contradictory results have been presented about the risk posed for recurrence.^40–43^ Data provided by the currently available studies was not sufficient to comment on any alteration of the timing of recurrence associated with diabetes. Many studies documented cavitation of the lung during the initial episode of PTB and non-resolved cavitation or residual cavitary disease following treatment completion as independent risk factors for recurrence.^24,28,41,44–46^ Daily DOT reduced the risk of relapse by 15.8% compared to standard thrice weekly treatment.^24,35,47,48^ When patients were treated with RIF and INH only instead of the current standard four anti-TB drugs, the risk of TB recurrence increased 2.5-fold compared to patients treated with the standard therapy of 6 months.^44^ Initial drug resistance, particularly to INH and RIF, or non-use of PZA with a shorter duration of RIF therapy, were risk factors leading to relapse and failure.^27,45,49^ The emergence of resistance during treatment was generally uncommon.

Interferon-γ, interleukin-12, and tumour necrosis factor α (TNF-α) pathways contribute to the control of infection by mycobacteria.^50,51^ Adult-onset immunodeficiency associated with anti-interferon autoantibodies is a risk factor for severe mycobacterial infections,^52,53^ but no studies have directly addressed subtle or profound deficits in these pathways and any association with relapse or reinfection.

Effective anti-TB drugs were first introduced in the 1940s, and combination drug treatment has been able to cure most TB patients since the late 1960s. The overall successful treatment rate of PTB remains at ∼85%, but for MDR-TB, it is only 57%. The first-line anti-TB drugs, RIF, INH, PZA, and ethambutol, are roughly adjusted to the total body weight of the patient before administration.^54,55^ Up to 10-fold variation of anti-TB drug exposure between patients has been noted; suboptimal concentrations of anti-TB drugs are significantly associated with poorer treatment outcomes, particularly treatment failure and development of drug resistance.^54,56–63^ Therapeutic drug monitoring contributes to optimising treatment outcomes and reducing the development of drug resistance.^55^ None of the studies identified in this review addressed this issue.

Clinical confusion with recurrent PTB may be due to many lung disorders, including exacerbations of chronic obstructive pulmonary disease (COPD), asthma, pneumonia, and post-TB bronchiectasis, resulting in misdiagnosis and incorrect management, increasing morbidity and mortality. In high-burden countries with pulmonary TB, the main reason for bronchiectasis is post-TB.^64–71^ In terms of the timeframe of relapse of PTB and post-TB bronchiectasis, a marked difference was not identified by the literature available. Post-tuberculous COPD is an indicator of a major reduction in lung function after PTB linked to breathlessness on exertion.^72,73^

Radiological confusion with recurrent PTB is most common with chronic pulmonary aspergillosis (CPA), which is a complication following PTB (especially in those with residual cavitation) and is frequently misdiagnosed as relapsed PTB.^74–77^ Besides being similar radiologically, these people are not clinically distinguishable either, although fever is more common with PTB and significant haemoptysis more prominent with CPA.^77^ In studies in Vietnam, India, and Ghana, over 50% of patients re-presenting with symptoms of possible PTB after a cure had CPA.^76,78,79^ In many high TB burden countries, patients with possible recurrent PTB present with similar respiratory symptoms and are commonly diagnosed as relapsed PTB even when sputum acid-fast bacilli smear or GeneXpert *M. tuberculosis* are negative.^80–82^ Smear-negative cases should alert clinicians to the possibility of CPA.^82,83^ In Ghana, Ocansey et al. conducted a study and found that CPA was often misdiagnosed as a relapse of PTB.^79^ They pointed out that when a patient presents with symptoms suspicious of TB relapse following an initial episode of PTB, CPA should be one of the top differentials, particularly in high-endemic areas for TB and CPA. A study conducted in Vietnam, a country with a high TB burden, recorded the time interval from PTB to CPA. The interval between the diagnosis of PTB and CPA was under 5 years in 27.3%, with 30.3% presenting 5 to 10 years later and 42.4% after 10 years.^78^ Similarly, another study from Brazil documented that the median time from the first diagnosis of PTB to the diagnosis of aspergilloma with severe haemoptysis was 9 years.^84^ Therefore, the time interval of relapsed PTB is significantly shorter than the time interval to develop CPA following PTB cure. Suspicion of CPA is crucial when considering PTB recurrence, particularly ≥2 years from PTB treatment completion. Chronic pulmonary histoplasmosis is another similar differential diagnosis.^85^ A positive CPA diagnosis can prevent patients from being unnecessarily exposed to anti-TB medications and should result in better patient outcomes.

When there is a clinical suspicion of CPA, a computerised tomography scan is used as a diagnostic aid tool as fungal balls (aspergillomas) are twice as frequently found compared with chest X-rays.^78,86–88^ *Aspergillus* immunoglobulin G is the simplest and most sensitive means of diagnosing CPA, with studies reporting 80–90% sensitivity.^80^ CPA and PTB may coexist, but RIF should not be given with azole antifungal as the drug interaction renders antifungal therapy useless. Earlier CPA diagnosis and treatment will reduce the high morbidity and mortality rates.^74,90–92^

Recently developed molecular typing methods are able to distinguish exogenous *M. tuberculosis* reinfection from endogenous re-activation.^93,94^ According to Shao et al., different genomic-based typing methods, such as MIRU-VNTR, IS*6110* fingerprinting, spoligotyping, and whole genome sequencing, have different discriminatory power, which determines the discrimination ability between various types of recurrences.^95^ GeneXpert MTB/RIF (*M. tuberculosis*/RIF) is mainly utilised for RIF-resistant screening. It delivers false-positive results, mainly in low *M. tuberculosis* load.^96,97^ When GeneXpert is used following a longer time period since the cure of PTB, it may produce more accurate results.^98^

Lack of data or misclassification of relapse and reinfection was a limitation we identified.^98–101^ Many studies lacked details on deaths and loss to follow-up of the patients. One of the strengths of this study was the high number of patients included in the study, allowing us to recognise true patterns of recurrences, relapses, and reinfections. None of the individual studies were able to assess large numbers of patients. Another strength identified was that all the patients included in our study had confirmed TB and completed adequate treatment.

Reducing relapse speaks to optimising PTB therapy to improve the efficacy of the treatment for the first drug-susceptible episode, while the relatively high proportion of reinfection in high-burden countries shows the importance of reducing the risk of TB transmission.^102,103^ With ∼1 in 35 patients having a recurrence, and most of these relapses occurring within 24 months, an argument can be made for follow-up for at least 2 years after treatment completion.^104^ This argument is strengthened by the frequent complication of CPA in PTB (10–13%) within 2 years of PTB treatment completion.

In conclusion, our findings demonstrate that PTB recurrences closer to treatment completion are mainly relapses, while late recurrences are more likely to be reinfections. In low TB prevalent settings, relapse is more common, while the contribution from both relapse and reinfection is similar in high TB prevalent settings. Patients who complete TB treatment should be followed up for at least 24 months to detect potential relapse or complications. Analysis of the timeframe of PTB recurrence enables clinical differentiation from other diseases with similar presentation, even in a low-resource setting.

## Supplementary Material


