# Supplementary material for: Timing of recurrence after treatment of pulmonary TB
Source: IJTLD Open. 2024 Oct 1;1(10):456–65. doi: 10.5588/ijtldopen.24.0222 (PMC11467853; doi:10.5588/ijtldopen.24.0222)
Supplement: Supplementary file 1 [file ijtldopen24-0222_Supplementarydata1.doc]

**SUPPLEMENTARY DATA**

# External quality assurance of chest X-ray interpretation to strengthen diagnosis of childhood TB

**Timing of recurrence after treatment of pulmonary tuberculosis**

Supplementary Figure S1: Flow diagram - Literature search and screening process


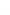

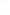

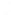


Published articles identified

through database searching (2627)

Articles removed

because of foreign languages (18)

Articles removed because

of duplication (275)

Articles screened by

abstracts and titles (2330)

Articles excluded

(2082)

Full articles assessed for

eligibility (248)

Russian articles did not

match inclusion criteria (12)

Articles did not have a clear time-frame (38)

Studies included in

systematic review (23)

Diagnosis clinically and by

chest x-rays (4)

Articles removed due to

other reasons (181)


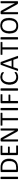

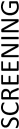

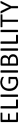

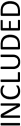


**References included in Table 1 and studies included in the systematic review.**

Caminero JA, Pena MJ, Campos-Herrero MI, Rodríguez JC, Afonso O, Martin C, Pavón JM, Torres MJ, Burgos M, Cabrera P, Small PM, Enarson DA. Exogenous reinfection with tuberculosis on a European island with a moderate incidence of disease. Am J Respir Crit Care Med. 2001 Mar;163(3 Pt 1):717-20. doi: 10.1164/ajrccm.163.3.2003070. PMID: 11254530

Cacho J, Pérez Meixeira A, Cano I, Soria T, Ramos Martos A, Sánchez Concheiro M, Samper S, Gavin P, Martín C. Recurrent tuberculosis from 1992 to 2004 in a metropolitan area. Eur Respir J. 2007 Aug;30(2):333-7. doi: 10.1183/09031936.00005107. PMID: 17504801

Johnson JL, Okwera A, Nsubuga P, Nakibali JG, Whalen CC, Hom D, Cave MD, Yang ZH, Mugerwa RD, Ellner JJ. Efficacy of an unsupervised 8-month rifampicin-containing regimen for the treatment of pulmonary tuberculosis in HIV-infected adults. Uganda-Case Western Reserve University Research Collaboration. Int J Tuberc Lung Dis. 2000 Nov;4(11):1032-40. PMID: 11092715

Uys P, Brand H, Warren R, van der Spuy G, Hoal EG, van Helden PD. The Risk of Tuberculosis Reinfection Soon after Cure of a First Disease Episode Is Extremely High in a Hyperendemic Community. PLoS One. 2015 Dec 9;10(12):e0144487. doi: 10.1371/journal.pone.0144487. PMID: 26649422

Pascopella L, Deriemer K, Watt JP, Flood JM. When tuberculosis comes back: who develops recurrent tuberculosis in California? PLoS One. 2011;6(11):e26541. doi: 10.1371/journal.pone.0026541. PMID: 22069456

Dobler CC, Marks GB, Simpson SE, Crawford AB. Recurrence of tuberculosis at a Sydney chest clinic between 1994 and 2006: reactivation or reinfection? Med J Aust. 2008 Feb 4;188(3):153-5. doi: 10.5694/j.1326-5377.2008.tb01558.x. PMID: 18241171

OR

Dobler CC, Crawford AB, Jelfs PJ, Gilbert GL, Marks GB. Recurrence of tuberculosis in a low-incidence setting. Eur Respir J. 2009 Jan;33(1):160-7. doi: 10.1183/09031936.00104108. PMID: 18829676

Sonnenberg P, Murray J, Glynn JR, Shearer S, Kambashi B, Godfrey-Faussett P. HIV-1 and recurrence, relapse, and reinfection of tuberculosis after cure: a cohort study in South African mineworkers. Lancet. 2001 Nov 17;358(9294):1687-93. doi: 10.1016/S0140-6736(01)06712-5. PMID: 11728545

Bandera A, Gori A, Catozzi L, Degli Esposti A, Marchetti G, Molteni C, Ferrario G, Codecasa L, Penati V, Matteelli A, Franzetti F. Molecular epidemiology study of exogenous reinfection in an area with a low incidence of tuberculosis. J Clin Microbiol. 2001 Jun;39(6):2213-8. doi: 10.1128/JCM.39.6.2213-2218.2001. PMID: 11376059

Crampin AC, Mwaungulu JN, Mwaungulu FD, Mwafulirwa DT, Munthali K, Floyd S, Fine PE, Glynn JR. Recurrent TB: relapse or reinfection? The effect of HIV in a general population cohort in Malawi. AIDS. 2010 Jan 28;24(3):417-26. doi: 10.1097/QAD.0b013e32832f51cf. PMID: 20042847

Jasmer RM, Bozeman L, Schwartzman K, Cave MD, Saukkonen JJ, Metchock B, Khan A, Burman WJ; Tuberculosis Trials Consortium. Recurrent tuberculosis in the United States and Canada: relapse or reinfection? Am J Respir Crit Care Med. 2004 Dec 15;170(12):1360-6. doi: 10.1164/rccm.200408-1081OC. PMID: 15477492

Marx FM, Dunbar R, Enarson DA, Williams BG, Warren RM, van der Spuy GD, van Helden PD, Beyers N. The temporal dynamics of relapse and reinfection tuberculosis after successful treatment: a retrospective cohort study. Clin Infect Dis. 2014 Jun;58(12):1676-83. doi: 10.1093/cid/ciu186. PMID: 24647020

Glynn JR, Yates MD, Crampin AC, Ngwira BM, Mwaungulu FD, Black GF, Chaguluka SD, Mwafulirwa DT, Floyd S, Murphy C, Drobniewski FA, Fine PE. DNA fingerprint changes in tuberculosis: reinfection, evolution, or laboratory error? J Infect Dis. 2004 Sep 15;190(6):1158-66. doi: 10.1086/423144. PMID: 15319867

Chang KC, Leung CC, Yew WW, Ho SC, Tam CM. A nested case-control study on treatment-related risk factors for early relapse of tuberculosis. Am J Respir Crit Care Med. 2004 Nov 15;170(10):1124-30. doi: 10.1164/rccm.200407-905OC. PMID: 15374844

Hung CL, Chien JY, Ou CY. Associated factors for tuberculosis recurrence in Taiwan: a nationwide nested case-control study from 1998 to 2010. PLoS One. 2015 May 1;10(5):e0124822. doi: 10.1371/journal.pone.0124822. PMID: 25932917

Narayanan S, Swaminathan S, Supply P, Shanmugam S, Narendran G, Hari L, Ramachandran R, Locht C, Jawahar MS, Narayanan PR. Impact of HIV infection on the recurrence of tuberculosis in South India. J Infect Dis. 2010 Mar;201(5):691-703. doi: 10.1086/650528. PMID: 20121433

Thomas A, Gopi PG, Santha T, Chandrasekaran V, Subramani R, Selvakumar N, Eusuff SI, Sadacharam K, Narayanan PR. Predictors of relapse among pulmonary tuberculosis patients treated in a DOTS programme in South India. Int J Tuberc Lung Dis. 2005 May;9(5):556-61. PMID: 15875929

Vieira AA, Leite DT, Adreoni S. Tuberculosis recurrence in a priority city in the state of Sao Paulo, Brazil. J Bras Pneumol. 2017 Mar-Apr;43(2):106-112. doi: 10.1590/S1806-37562016000000002. PMID: 28538777

Moosazadeh M, Bahrampour A, Nasehi M, Khanjani N. The incidence of recurrence of tuberculosis and its related factors in smear-positive pulmonary tuberculosis patients in Iran: A retrospective cohort study. Lung India. 2015 Nov-Dec;32(6):557-60. doi: 10.4103/0970-2113.168113. PMID: 26664159

Youn HM, Shin MK, Jeong D, Kim HJ, Choi H, Kang YA. Risk factors associated with tuberculosis recurrence in South Korea determined using a nationwide cohort study. PLoS One. 2022 Jun 16;17(6):e0268290. doi: 10.1371/journal.pone.0268290. eCollection 2022. PMID: 35709199

Ruan QL, Yang QL, Sun F, Liu W, Shen YJ, Wu J, Jiang N, Zhou JY, Shao LY, Zhang WH. Recurrent pulmonary tuberculosis after treatment success: a population-based retrospective study in China. Clin Microbiol Infect. 2022 May;28(5):684-689. doi: 10.1016/j.cmi.2021.09.022. PMID: 34601149

Shao Y, Song H, Li G, Li Y, Li Y, Zhu L, Lu W, Chen C. Relapse or Re-Infection, the Situation of Recurrent Tuberculosis in Eastern China. Front Cell Infect Microbiol. 2021 Mar 17;11:638990. doi: 10.3389/fcimb.2021.638990. eCollection 2021. PMID: 33816342

He W, Tan Y, Song Z, Liu B, Wang Y, He P, Xia H, Huang F, Liu C, Zheng H, Pei S, Liu D, Ma A, Cao X, Zhao B, Ou X, Wang S, Zhao Y. Endogenous relapse and exogenous reinfection in recurrent pulmonary tuberculosis: A retrospective study revealed by whole genome sequencing. Front Microbiol. 2023 Feb 17;14:1115295. doi: 10.3389/fmicb.2023.1115295. PMID: 36876077

Pamra SP, Prasad G, Mathur GP. Relapse in pulmonary tuberculosis. Am Rev Respir Dis. 1976 Jan;113(1):67-72. doi: 10.1164/arrd.1976.113.1.67.

Supplementary Figure S2A: Kaplan-Meier curve. Comparison of time interval for recurrence to occur between HIV positive and HIV negative patient groups.


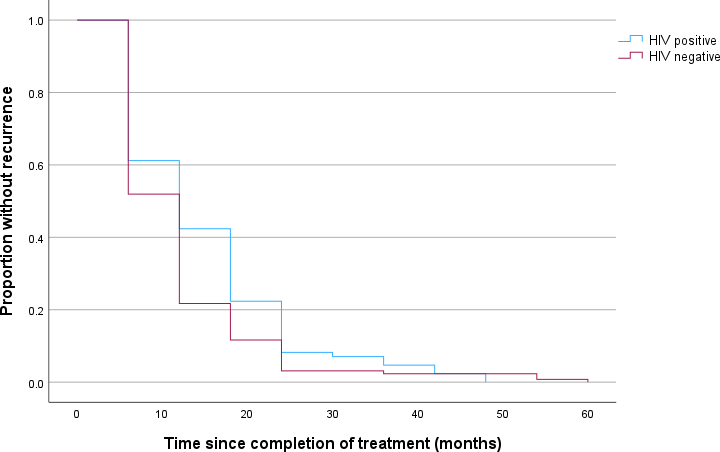


P=0.038

chi-square=4.303

Supplementary Figure S2B: Kaplan-Meier curve. Comparison of time interval for relapse in HIV positive and HIV negative patient groups.


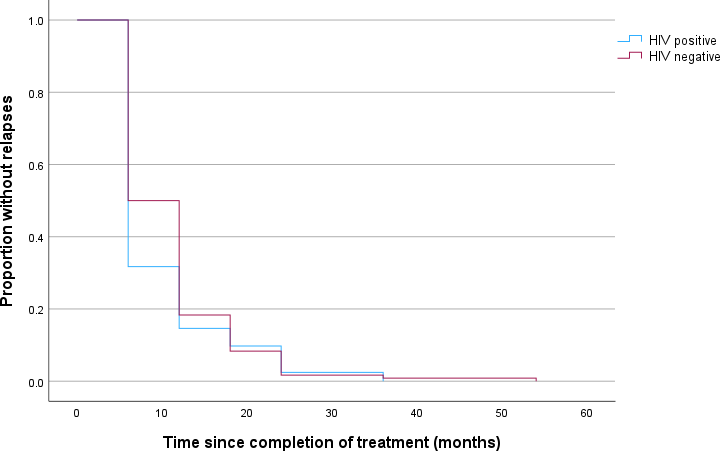


p=0.280

chi-square=1.168

Supplementary Figure S2C: Comparison of time interval for reinfection between HIV positive and HIV negative patient groups.


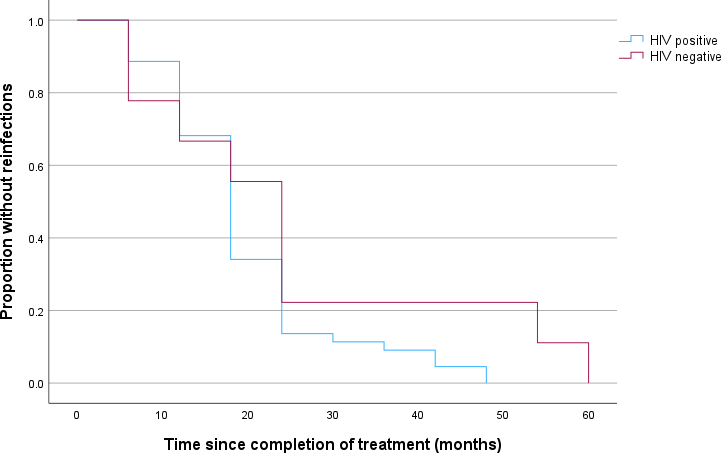


p=0.117

chi-square=2.463
